# Neuroinflammatory regulatory role of microglia in optic nerve injury: from pathological mechanisms to therapeutic targets

**DOI:** 10.3389/fimmu.2026.1742677

**Published:** 2026-02-02

**Authors:** Miaoran Gao, Jian Zhou, Nannan Shi, Xiaoling Yan, Lina Liang

**Affiliations:** 1 Eye Hospital, China Academy of Chinese Medical Sciences, Beijing, China; 2 Postdoctoral Research Station of China Academy of Chinese Medical Sciences, Beijing, China; 3 Department of Ophthalmology, Dongfang Hospital, Beijing University of Chinese Medicine, Beijing, China

**Keywords:** axonal regeneration, microglia, microglial polarization, neuroinflammation, optic nerve injury, therapeutic targets

## Abstract

Optic nerve injury, encompassing conditions such as glaucoma, optic neuritis, and traumatic optic neuropathy, is a major cause of irreversible vision loss. Traditional broad-spectrum anti-inflammatory treatments have shown limited efficacy, highlighting the need for precision-based therapeutic approaches grounded in the underlying pathological mechanisms. As the primary immune cells of the central nervous system (CNS), microglia play a crucial role in regulating neuroinflammation following optic nerve injury. This review provides a comprehensive overview of the mechanisms governing microglial neuroinflammatory regulation, including early inflammatory signal recognition mediated by damage-associated molecular patterns (DAMPs), dynamic transcriptional networks regulating polarization between pro-inflammatory and pro-repair phenotypes, bidirectional modulation of phagocytic clearance by the complement system, and the complex multicellular interactions among microglia, astrocytes, and Müller cells. The concept of conditioning injury conditioning injury (intraocular inflammatory stimulation) has revealed the dual nature of neuroinflammatory responses: through temporal polarization shifts, microglia can both release neurotoxic mediators that worsen injury and secrete neurotrophic factors that promote axonal regeneration and myelin repair. This shift from traditional broad-spectrum anti-inflammatory strategies to precision functional modulation forms the basis for emerging therapeutic approaches, including PPARγ pathway activation, selective complement system targeting, and time-dependent modulation. We also assess the potential of advanced technologies, such as nanodelivery systems, single-cell analysis, and molecular imaging, in precision diagnosis and treatment. Finally, we critically examine the limitations of current research, including interspecies variability, model constraints, and clinical translation barriers, and discuss the translational potential of microglia-targeted therapies in protecting and restoring clinically meaningful visual function.

## 1 Introduction

Optic nerve injury encompasses a range of conditions, including glaucoma, optic neuritis, ischemic optic neuropathy, optic pathway gliomas, and traumatic optic neuropathy, all of which are characterized by retinal degeneration (1, 2). The global burden of these conditions is substantial: glaucoma affects approximately 76 million people worldwide and is the second leading cause of blindness (3); traumatic optic neuropathy occurs in 0.5–5% of cases of craniocerebral trauma (4, 5); and optic neuritis has an annual incidence of 1–5 per 100,000 individuals, with half of these patients at risk of developing multiple sclerosis (6). Optic pathway gliomas occur in approximately 15–20% of children with neurofibromatosis type 1 and can lead to progressive vision loss (7). Despite the high prevalence of these disorders, current treatments remain largely ineffective, leaving many patients with irreversible vision loss.

The limitations of traditional broad-spectrum anti-inflammatory therapies have prompted a re-evaluation of the complexity of neuroinflammation in optic nerve injury. Recent discoveries regarding conditioning injury —where intraocular inflammation induced by lens injury or zymosan injection promotes axonal regeneration— have challenged the purely harmful view of inflammation, showing that under certain conditions, inflammatory responses can promote axonal regeneration (8). However, significant translational challenges arise due to key differences between species: human optic nerves are approximately 25 times longer and contain 30 times more retinal ganglion cells (RGCs) than those of mice, and adult human RGCs exhibit substantially lower regenerative capacity, which further declines with age (9–11). These species differences highlight the need for a mechanistic understanding of neuroinflammatory regulation.

As the primary immune cells of the central nervous system (CNS), microglia play a pivotal role in orchestrating neuroinflammatory responses following optic nerve injury (12, 13). Advances in understanding the mechanisms of microglial M1/M2 polarization switching, bidirectional complement system regulation, and glial cell interaction networks (14–17) have revealed the positive regulatory role of neuroinflammation in injury resolution, microenvironmental homeostasis, and the initiation of endogenous repair programs. The M1/M2 polarization framework, though conceptually useful, oversimplifies microglial biology. Microglial responses are dynamic and context-dependent, not binary. Field-wide consensus now recognizes this complexity (18). This recognition has transformed therapeutic approaches. Strategies now focus on precision modulation. This approach enhances beneficial inflammatory functions while constraining harmful ones. This shift in understanding drives the transition from broad-spectrum anti-inflammatory suppression to precision functional modulation, which harnesses beneficial inflammatory responses while mitigating harmful ones. This review systematically examines the current understanding of microglial neuroinflammatory regulation in optic nerve injury, evaluates strategies for functional inflammation modulation, and discusses their clinical translation prospects, while critically assessing research limitations to provide a theoretical foundation for the development of precision treatments.

## 2 Mechanisms of neuroinflammatory regulation by microglia

**Figure 1:**
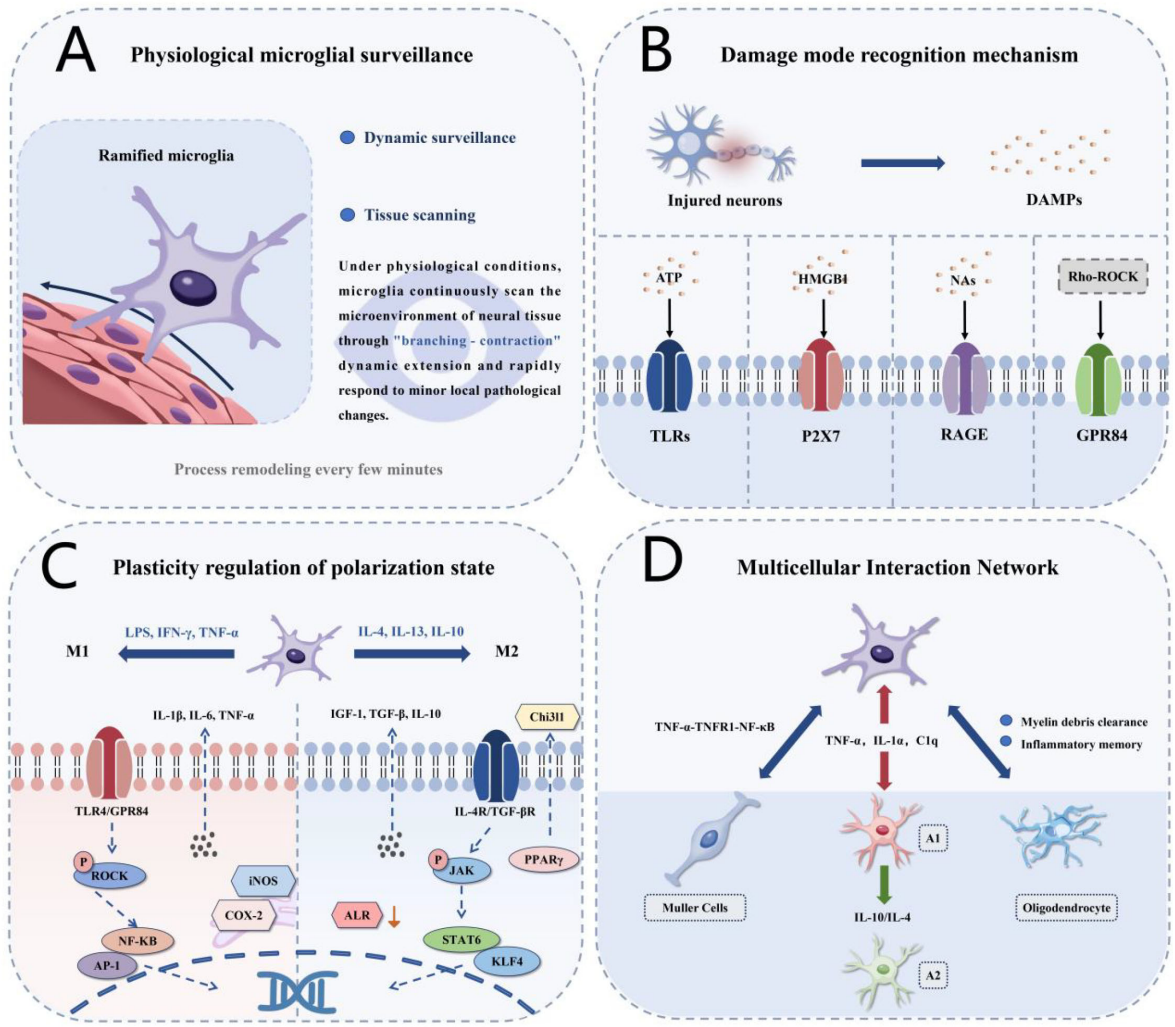
Molecular mechanisms of microglial surveillance and multicellular inflammatory networks in the optic nerve. **(A)** Resting microglia with ramified morphology performing tissue surveillance. **(B)** DAMP recognition through pattern recognition receptors (TLRs, P2X7R, RAGE, GPR84). **(C)** M1/M2 polarization pathways with key transcription factors and markers, including the GPR84-rho-ROCK pathway and aldose reductase signaling. **(D)** Multicellular networks involving astrocytes (A1/A2 phenotypes), Müller cells, and oligodendrocytes with bidirectional signaling arrows showing temporal dynamics. M1/M2 labels indicate pro-inflammatory/pro-repair phenotypes, recognizing these represent a functional continuum.

As key immune effector cells of the CNS, microglia orchestrate neuroinflammatory responses through three critical mechanisms: detecting injury signals via pattern recognition receptor systems and initiating graded inflammatory responses; dynamically shifting between pro-inflammatory and pro-repair functional states in response to microenvironmental signals; and establishing multicellular interaction networks with astrocytes and Müller cells, which collectively determine the intensity and outcome of neuroinflammation (**Figure 1****).**

### 2.1 Molecular basis of inflammatory surveillance and activation

Microglia possess distinct inflammatory surveillance capabilities, relying on precise molecular recognition mechanisms and dynamic morphological plasticity. Under normal physiological conditions, microglia constantly monitor tissue microenvironments through surface pattern recognition receptors, maintaining dynamic patrolling states in which their processes remodel every few minutes, thereby conducting comprehensive regional surveillance within hours (19, 20). This surveillance allows for the rapid detection of pathogen-associated molecular patterns (PAMPs) and damage-associated molecular patterns (DAMPs), thereby triggering inflammatory responses (21). Microglial activation exhibits clear threshold effects: minor disturbances induce localized morphological changes, while intense injury signals activate extensive inflammatory programs (22).

Following optic nerve injury, damaged neurons release abundant endogenous DAMPs, such as ATP, HMGB1, and nucleic acid fragments, which bind to TLRs, P2 receptors, and RAGE, initiating inflammatory transcriptional programs (23–25). Within hours, microglia exhibit typical activation features, including cell body enlargement, process retraction, and transformation from a ramified to an amoeboid morphology, accompanied by directional migration toward injury sites and the release of pro-inflammatory factors (26). This DAMP-mediated activation forms the molecular basis for the transition from surveillance to an inflammatory state.

### 2.2 Transcriptional regulatory networks regulating microglial polarization

The M1/M2 polarization framework, while conceptually useful, represents a simplified view of microglial biology that recent field-wide consensus has critically re-evaluated (18). Emerging evidence demonstrates that microglial activation exists along a functional continuum rather than as discrete binary states, with individual cells capable of exhibiting mixed phenotypic features that shift dynamically in response to temporal progression, spatial gradients, and local microenvironmental signals (27, 28). Single-cell transcriptomic analyses have revealed that molecular signatures alone do not deterministically predict functional outcomes—transcriptionally similar microglia may execute divergent biological programs depending on broader tissue context (29, 30). This disconnect between transcriptional state and functional output emphasizes that therapeutic strategies cannot simply target ‘M1’ or ‘M2’ phenotypes, but must instead modulate specific functional capacities within their relevant spatiotemporal contexts (31). Nevertheless, understanding the signaling pathways that bias microglial responses toward pro-inflammatory or pro-repair trajectories provides valuable mechanistic insight for rational therapeutic design.

Microglia attain distinct functional states through complex transcriptional regulatory networks that govern pro-inflammatory and pro-repair polarization (32). Polarization toward pro-inflammatory phenotypes (M1-like) is induced by pro-inflammatory signals, including LPS, IFN-γ, and TNF-α, with transcription factors such as NF-κB, AP-1, and IRF synergistically upregulating pro-inflammatory genes like iNOS, COX-2, and CD86, thereby promoting the secretion of IL-1β, IL-6, and TNF-α (33–35). These Microglia exhibiting pro-inflammatory phenotypes undergo metabolic reprogramming from oxidative phosphorylation to glycolysis, facilitating the rapid generation of ATP and pro-inflammatory mediators (36). Although M1 and M2 represent oversimplified categories, understanding the signaling pathways that bias microglial responses provides mechanistic insight. Recent studies have identified additional signaling pathways that regulate pro-inflammatory phenotypes, including GPR84-mediated rho-ROCK pathway activation and aldose reductase as an inflammatory mediator, with their inhibition demonstrating neuroprotective effects in optic nerve injury models (37, 38). Conversely, polarization toward pro-repair phenotypes (M2-like) is induced by anti-inflammatory signals such as IL-4, IL-13, and IL-10, with transcription factors like STAT6, KLF4, and PPARγ promoting the expression of anti-inflammatory genes such as Arg1, CD206, and Chi3l1 (39, 40). Microglia with pro-repair characteristics maintain dendritic morphology and secrete anti-inflammatory factors, including TGF-β and IL-10, as well as neurotrophic factors like BDNF and IGF-1, sustaining their function through mitochondrial oxidative phosphorylation and fatty acid oxidation (41, 42). The phenotypic states are dynamic processes controlled through transcriptional regulation, epigenetic modifications, and metabolic reprogramming (43). Facial nerve injury models show that bipolar or rod-shaped cells rapidly transform to amoeboid morphology following LPS stimulation, with a marked upregulation of pro-inflammatory markers, highlighting the remarkable plasticity of microglial polarization (44). This plasticity provides the biological foundation for therapeutic interventions targeting the switching of polarization states. Beyond biochemical signals, emerging evidence suggests that glial cells may respond to mechanical cues through mechanosensitive ion channels such as Piezo1, which can influence cellular calcium signaling and functional states (45). Substrate stiffness increases following CNS injury due to glial scar formation (46), and such mechanical changes have been shown to modulate immune cell activation in other contexts, potentially contributing to microglial phenotypic plasticity in optic nerve injury.

### 2.3 Regulatory mechanisms of multicellular interaction networks

Neuroinflammation represents a complex network system involving the coordinated participation of microglia, astrocytes, Müller cells, and oligodendrocytes. The dynamic balance of this network directly determines the intensity, duration, and pathological outcome of inflammatory responses. Microglia-astrocyte interactions are a core element of this network: activated microglia induce astrocyte transformation into the neurotoxic A1 phenotype through the secretion of TNF-α, IL-1α, and C1q (16). These A1 astrocytes lose their neuroprotective functions and instead secrete neurotoxic factors, creating pathological positive feedback loops with activated microglia that amplify inflammatory responses. However, the toxic effects of the A1 phenotype exhibit spatiotemporal specificity, as these cells may contribute to early injury clearance and exhibit neurotoxicity only under chronic activation (47). Recent evidence shows that astrocyte polarization follows distinct temporal dynamics during chronic optic nerve injury, with protective A2 phenotypes emerging within 2–8 weeks of elevated intraocular pressure, while detrimental A1 polarization occurs only after 8 weeks. This suggests a potential therapeutic window before neurotoxic responses dominate (48).

In the retinal environment, Müller cells add complexity to the network by performing dual functions: maintaining retinal homeostasis under physiological conditions and potentially amplifying inflammation through TNF-α–TNFR1–NF-κB signaling or initiating endogenous repair programs under appropriate stimulation (49). In glaucomatous injury, microglial TNF-α induces Müller cell activation via TNFR1–NF-κB signaling. Activated Müller cells then secrete additional pro-inflammatory factors, establishing sustained inflammatory cycles that promote retinal ganglion cell (RGC) apoptosis (50). Oligodendrocyte injury plays a critical role in the chronicity of neuroinflammation, as damaged oligodendrocytes release myelin debris as sustained endogenous danger signals that activate microglia and may establish inflammatory memory through epigenetic modifications (51). This concept of trained immunity suggests that innate immune cells acquire enhanced responsiveness through epigenetic reprogramming, potentially explaining sustained neuroinflammatory responses following initial injury, even after the elimination of pathogenic factors. The microglia-astrocyte interaction also contributes to glial scar formation, which creates a physical barrier to axonal regeneration. Reactive astrocytes deposit chondroitin sulfate proteoglycans (CSPGs) that inhibit axon extension, while microglial cytokines (TNF-α, IL-1α, C1q) promote astrogliosis, and astrocyte-derived factors sustain microglial activation, establishing a self-perpetuating barrier. This bidirectional signaling represents a critical therapeutic target for enhancing regeneration (52).

### 2.4 Single-cell transcriptomic insights into microglial heterogeneity

Single-cell RNA sequencing has revealed unexpected complexity in microglial responses to optic nerve injury. Instead of transitioning between two distinct activation states, microglia adopt multiple specialized transcriptional programs, each associated with distinct roles in injury clearance, inflammation regulation, and tissue repair.

#### 2.4.1 Disease-associated microglia in the injured optic nerve

Single-cell RNA sequencing studies have identified distinct microglial populations in glaucoma and retinal degeneration models. Some populations are preferentially enriched in the optic nerve compared to the retina (53, 54). These activated microglia exhibit molecular features characteristic of disease-associated microglia (DAM) in Alzheimer’s disease. Key features include elevated expression of TREM2, APOE, and genes regulating lipid metabolism.

DAM activation proceeds through two sequential stages. The initial TREM2-independent phase involves downregulation of homeostatic markers (P2ry12, Tmem119) and upregulation of APOE. The subsequent TREM2-dependent phase enables full phagocytic capacity via increased expression of lysosomal and lipid-processing genes (LPL, CST7, AXL) (55). Functional studies confirm that TREM2 signaling is essential for microglial recruitment and phagocytic clearance of degenerating photoreceptors. TREM2 deficiency exacerbates neuronal loss in retinal degeneration models (56, 57). Collectively, these findings indicate that DAM function as protective responders during acute injury, specializing in removal of toxic breakdown products.

#### 2.4.2 Temporal evolution of microglial subpopulations

Microglial responses to nerve injury evolve dynamically through temporally defined phases. Single-cell RNA sequencing throughout the mouse lifespan and following brain injury has identified transient microglial subpopulations with distinct gene expression profiles (28). Recovery-associated clusters exhibit elevated APOE expression and emerge specifically during tissue resolution phases. These subsets appear at defined time points after injury and differ from both homeostatic and acutely activated microglia. This temporal specificity suggests specialized functions in restoring CNS homeostasis.

Temporal dynamics also govern microglial proliferation. Nerve injury triggers microglial expansion through local proliferation and potential recruitment. This process occurs in distinct waves characterized by coupled proliferation and apoptosis (58, 59). The functional significance of these temporally segregated populations remains incompletely understood. However, they likely reflect shifting tissue demands, including debris clearance, scar formation, and tissue remodeling support.

#### 2.4.3 Implications for selective therapeutic modulation

Microglial heterogeneity enables precision interventions that enhance beneficial subpopulations while limiting detrimental ones. The TREM2-APOE pathway represents a promising therapeutic target. Endogenous pro-resolving lipid mediators, including resolvins and lipoxins, can modulate excessive microglial reactivity and promote neuroinflammation resolution (60, 61).

Alternative therapeutic approaches can engage downstream effectors when specific signaling pathways are compromised. PPARγ agonists enhance microglial phagocytic function and promote toxic debris clearance through CD36-mediated mechanisms (62). These agents offer strategies to restore beneficial microglial activities. However, timing is critical. Promoting DAM activation during acute injury may accelerate debris removal, whereas prolonged DAM persistence may drive chronic neuroinflammation. Effective clinical translation requires identifying optimal temporal windows when specific subpopulations benefit or impair recovery.

Despite recent advances, substantial knowledge gaps remain. Most single-cell studies have focused on glaucoma and retinal degeneration, with limited transcriptomic profiling of traumatic optic neuropathy. Spatial resolution presents another challenge. Bulk tissue profiling may obscure heterogeneity across anatomical subregions, including the injury epicenter, penumbra, and distal nerve. Integrating spatial transcriptomics with longitudinal sampling will be essential for mapping microglial state transitions across space and time. Such approaches will ultimately guide decisions regarding when and where to therapeutically intervene.

## 3 Neuroinflammatory cascade following optic nerve injury

**Figure 2:**
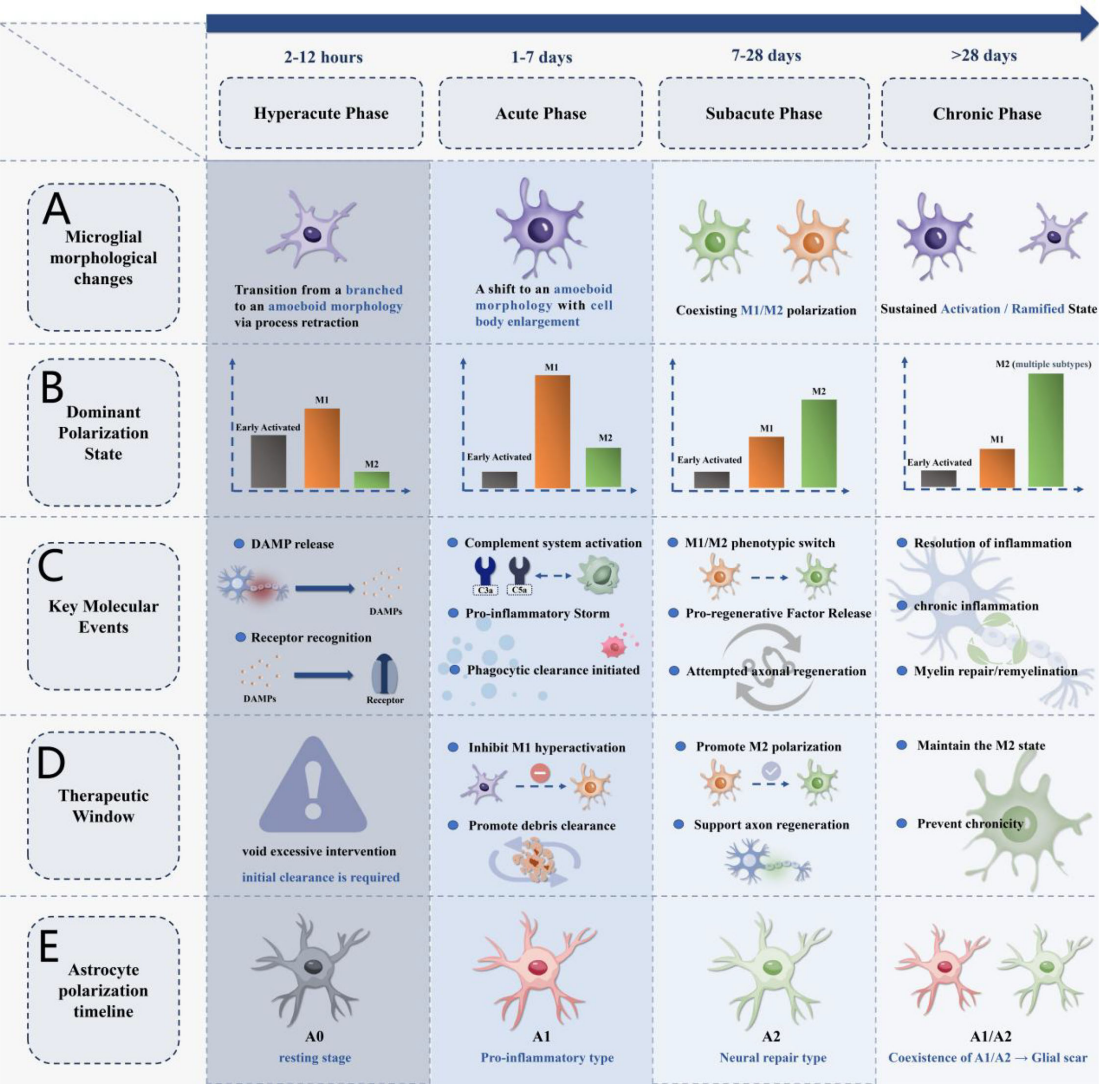
Spatiotemporal dynamics of neuroinflammation following optic nerve injury. **(A)** Microglial morphology changes from ramified (resting state) to amoeboid (activated state) during acute phase, with partial recovery in subacute and chronic phases. **(B)** Dominant polarization states showing M1 predominance during acute and subacute phases, with variable M1/M2 balance in chronic phase. **(C)** Key molecular events including DAMP release (HMGB1, ATP), complement activation (C1q, C3, MAC), and cytokine production (TNF-α, IL-1β, IL-6), with sustained complement component deposition during subacute phase. **(D)** Spatial gradient of inflammation intensity showing decreasing trend from injury epicenter to periphery. **(E)** Astrocyte polarization timeline demonstrating A2 (protective) phenotype emergence at 2 weeks and A1 (neurotoxic) phenotype appearance after 8 weeks of elevated intraocular pressure. Timeline and transitions represent schematic integration of data from cited references. M1/M2 labels indicate pro-inflammatory/pro-repair phenotypes, recognizing these represent a functional continuum.

Optic nerve injury triggers a highly organized neuroinflammatory cascade that progresses through distinct phases: DAMP release and recognition, complement-mediated phagocytic clearance, pro-inflammatory mediator synergy, and the formation of inflammatory responses with characteristic spatiotemporal patterns (**Figure 2**). Understanding the molecular mechanisms and spatiotemporal features of this cascade is critical for elucidating disease progression and developing targeted interventions.

### 3.1 DAMP-mediated early inflammatory signal recognition

The initiation of neuroinflammation following injury depends on the rapid recognition of DAMPs through the coordinated activation of multiple receptor pathways. Within hours post-injury, DAMPs released by damaged neural tissue activate local microglia and initiate inflammatory responses. HMGB1, a major nuclear-derived DAMP, activates MyD88–NF-κB signaling through TLR4 binding, promoting pro-inflammatory polarization (63). ATP released from damaged cells activates the purinergic receptor P2X7, triggering NLRP3 inflammasome assembly and promoting the maturation and release of IL-1β and IL-18 (64). Importantly, blockade of the P2X7 receptor has shown neuroprotective effects following optic nerve injury. Studies using the selective P2X7 antagonist A438079 or P2X7-deficient mice demonstrate delayed retinal ganglion cell loss after optic nerve crush, supporting P2X7 as a potential therapeutic target (65). Early complement system activation amplifies the inflammatory response through C3a generation, with this anaphylatoxin binding to C3aR1 and enhancing the inflammatory cascade (66). In glaucoma animal models, C3aR1 activation significantly promotes optic nerve degeneration, whereas C3ar1 gene-deficient mice exhibit pronounced neuroprotection (67). These coordinated DAMP recognition pathways form multiple signal input systems for microglial activation, ensuring rapid and precise inflammatory responses to neural injury.

### 3.2 Complement-mediated microglial phagocytic function

Microglial phagocytic function plays a central role in maintaining nervous system homeostasis and in pathological responses, achieving targeted clearance through complement-mediated molecular recognition. Following optic nerve injury, microglia clear synaptic debris and cellular remnants through phagocytosis, relying on the recognition of complement-generated opsonins (68). Complement activation cleaves C3 and C5, generating C3b and iC3b, which deposit on target cell surfaces as opsonins for CR3 recognition by microglia (69). Studies demonstrate that deficiencies in C1qa and integrin αM (CD11b) impair microglial phagocytic clearance capacity for neural tissue debris (70). Complement-mediated phagocytosis plays protective roles in neurodegenerative diseases; experimental glaucoma models show that intravitreal administration of human C1 inhibitor before and during elevated intraocular pressure preserves dendritic and synaptic integrity, potentially through C1q binding to apoptotic cells and promoting their phagocytosis by macrophages (71). CR3-mediated microglial phagocytosis is involved in various processes, including developmental synaptic pruning, pathological myelin clearance, amyloid-β clearance, and CNS pathogen elimination (72). Comprehensive analyses have substantiated the pathogenic role of complement dysregulation in glaucoma, showing that C1Q, C3, and membrane attack complex components critically mediate neuroinflammation and retinal ganglion cell death (73). Clinical studies indicate that decreased baseline serum levels of these complement components predict visual field loss progression in primary angle-closure glaucoma patients (74). Therapeutic complement modulation has advanced to clinical evaluation, with phase I trials demonstrating acceptable safety and target engagement of the anti-C1q antibody ANX007 in glaucoma patients (75).Beyond complement opsonization, microglia recognize apoptotic cells through the exposure of phosphatidylserine (PS) on the outer membrane leaflet. PS acts as an “eat-me” signal, recognized by the TAM receptor family (Tyro3, Axl, Mer) on microglial surfaces. The bridging molecules Gas6 and Protein S facilitate PS recognition by binding both PS and TAM receptors, thereby triggering phagocytosis. This PS-TAM pathway plays a crucial role in clearing apoptotic retinal ganglion cells following injury. However, it is a double-edged sword: while efficient debris clearance prevents secondary inflammation, premature phagocytosis of stressed but viable neurons may contribute to neuronal loss. Understanding the temporal regulation of PS exposure and TAM receptor expression is, therefore, critical for therapeutic intervention (76, 77).

### 3.3 Synergistic neurotoxic effects of pro-inflammatory mediators

Activated microglia release multiple pro-inflammatory mediators that interact through complex molecular networks, forming synergistic neurotoxic effects and establishing pathological positive feedback loops (78). TNF-α activates TNFR1 on neuronal surfaces, initiating the extrinsic apoptotic pathway and activating NF-κB, which promotes inflammatory gene transcription and amplifies feedback loops (79). IL-1β activates MyD88 signaling through IL-1R1 binding, directly damaging neurons while activating astrocytes and neighboring microglia, thereby expanding the inflammatory impact (80). IL-6 regulates inflammation- and cell survival-related genes through JAK/STAT3 pathway activation, contributing to the maintenance and chronification of neuroinflammation (81).

In addition to cytokines, reactive molecules such as NO and ROS contribute critically to neurotoxicity. Excessive NO and ROS production generates direct neurotoxicity: iNOS-produced NO directly damages neuronal DNA and proteins, combining with superoxide anions to form peroxynitrite (ONOO^−^), which causes lipid peroxidation, protein nitration, and mitochondrial dysfunction (82). These synergistic effects amplify the toxicity of individual mediators, establishing positive feedback networks that sustain neuroinflammation.

### 3.4 Spatiotemporal evolution patterns of neuroinflammation

Neural injury-triggered inflammatory responses exhibit highly organized spatiotemporal characteristics (83). The temporal progression includes four distinct stages: the hyperacute phase (2–12 hours), characterized by microglial morphological activation; the acute phase (1–7 days), marked by massive pro-inflammatory microglial activation and rapid pro-inflammatory mediator release; the subacute phase (7–28 days), representing a critical inflammatory transition period dominated by pro-inflammatory microglia; and the chronic phase (>28 days), during which inflammation either subsides with successful repair or establishes a persistent state (84, 85). Spatially, neuroinflammation shows layer-specific and regional gradient patterns, with post-injury microglial activation displaying a center-to-periphery gradient. Cell numbers are significantly elevated at injury centers, and inflammatory intensity within optic nerves decreases from injury sites to distal areas (86). Different microglial morphologies serve specific spatial functions; for example, rod-shaped cells specialize in clearing axonal debris (87). These precise spatiotemporal patterns reveal the intrinsic regulatory mechanisms of neuroinflammation and provide a theoretical foundation for developing precision therapeutic strategies based on temporal windows and spatial targeting.

## 4 Inflammatory regulatory role of microglia in axonal regeneration

**Figure 3:**
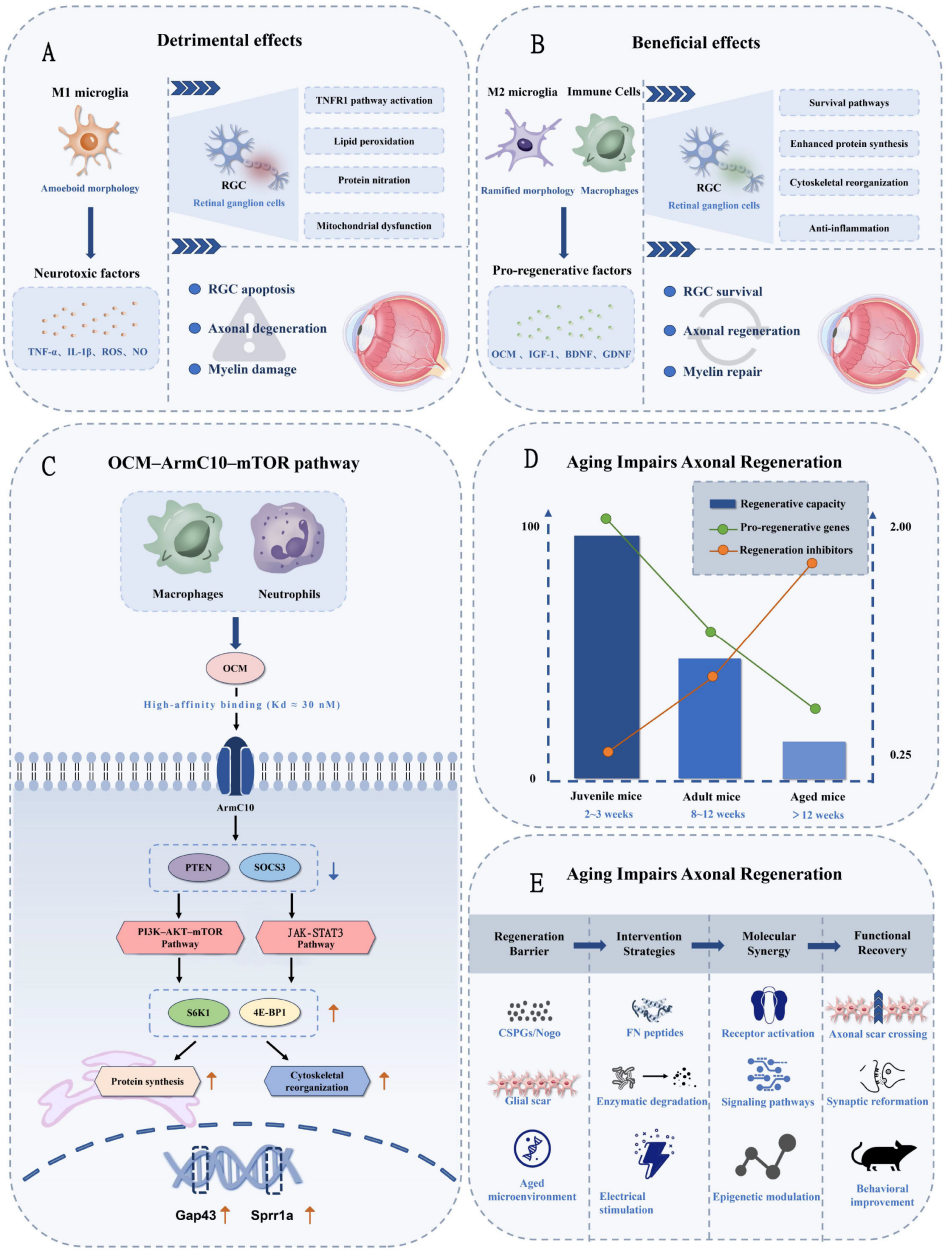
Dual regulatory roles of microglia in axonal regeneration and degeneration. **(A)** Detrimental effects - M1 microglia releasing neurotoxic factors (TNF-α, IL-1β, ROS) causing RGC death and axon degeneration. **(B)** Beneficial effects - M2 microglia and infiltrating immune cells producing pro-regenerative factors (OCM, IGF-1, BDNF) promoting axon regeneration. **(C)** Central panel showing the OCM-ArmC10-mTOR signaling pathway with PTEN/SOCS3 inhibition leading to enhanced protein synthesis and cytoskeletal reorganization. **(D)** Timeline showing age-dependent decline in regenerative capacity from juvenile to aged mice. **(E)** Therapeutic interventions including fibronectin peptides and multi-therapeutic approaches enhancing long-distance axonal regeneration.

Microglia exert condition-dependent regulatory effects on axonal regeneration, with their modes of action depending on the activation phase, intensity, and microenvironmental context. Conditioning injury-induced inflammatory activation promotes the production of pro-regenerative factors such as oncomodulin (OCM) by microglia and infiltrating immune cells, activating neuronal intrinsic regeneration programs through inhibition of PTEN–mTOR pathway negative regulation (11), with similar mechanisms validated in human iPSC-derived neurons (88). PTEN normally inhibits PI3K-AKT-mTOR by dephosphorylating PIP3; inflammatory signals reduce PTEN activity, releasing mTOR from this constraint. However, pro-regenerative effects exhibit significant age-dependence and species differences, and the double-edged nature of inflammatory responses poses clinical translation challenges regarding safety and timing control (9, 10) (**Figure 3**).

### 4.1 Pro-regenerative mechanisms of conditioning inflammatory activation

Conditioning injury effects represent an important discovery in neural regeneration research. Leon et al. first observed that lens injury promotes optic nerve axonal regeneration in adult rats (8). It is important to distinguish this ‘conditioning injury’ paradigm, specific to the optic nerve field, from the ‘preconditioning injury’ approach developed in spinal cord research. While both leverage inflammatory or injury-related signals to enhance regeneration, they differ mechanically and temporally. Peripheral nerve preconditioning (e.g., sciatic nerve transection 1–2 weeks before spinal cord injury) activates cell-autonomous growth programs within dorsal root ganglion neurons. In contrast, intraocular conditioning injury recruits immune cells that provide extracellular pro-regenerative signals. In the optic nerve context, the inflammation-dependent mechanism discovered by Leon et al. (8) showed that lens injury enhances RGC regeneration through macrophage-derived oncomodulin, establishing the foundation for subsequent mechanistic studies. Mechanistic studies revealed that during injury-induced inflammation, infiltrating neutrophils and macrophages rapidly express and secrete OCM, initiating pro-regenerative signal cascades upon binding to RGC surface receptors (89). Neutrophils rapidly infiltrate the eyes within 24–48 hours post-injury, expressing OCM and participating in inflammation-induced neural regeneration (90). The recent identification of the high-affinity OCM receptor ArmC10 revealed the central role of the OCM–ArmC10 ligand-receptor axis in conditioning injury effects (91).

Pro-regenerative effects exhibit pronounced age-dependent characteristics, with systematic studies across mouse age groups finding that juvenile mice (2–3 weeks) display strong regenerative responses, adult mice (8–12 weeks) show significantly attenuated effects, and aged mice (>12 months) possess extremely limited axonal regeneration capacity (92). This age-related decline involves multiple mechanisms: declining expression of RGC intrinsic regeneration-associated genes (Gap43, Sprr1a) with age, accumulation of inhibitory microenvironmental factors (CSPGs), and altered microglial responses to inflammatory stimuli with increased secretion of pro-inflammatory factors and decreased secretion of pro-regenerative factors (93, 94). Notably, partial visual function recovery in 12-month-old mice through epigenetic reprogramming suggests that age-related regeneration barriers may be partially reversible (93).At the earliest developmental stage, neonatal microglia exhibit unique regenerative capacity by orchestrating scar-free CNS repair through regulated extracellular matrix deposition. In contrast to adult microglia, which contribute to glial scar formation, neonatal microglia secrete controlled levels of fibronectin and TIMPs that promote organized tissue remodeling without fibrotic scarring. This microglia-dependent process, as evidenced by impaired healing following microglial depletion, suggests that rejuvenating adult microglia to adopt neonatal-like phenotypes may represent a potential therapeutic strategy (52).

The species translation of conditioning injury effects faces additional challenges due to significant anatomical differences between mouse and human optic nerve systems. Human optic nerves measure approximately 50 mm in total length with approximately 1.2 million RGC axons, whereas mouse optic nerves are only 2–5 mm long with approximately 55,000 RGCs (95). This scale difference means that human axons must regenerate over longer distances to reestablish functional connections, and differences in the degree of myelination, glial scar formation patterns, and inflammatory response duration may affect the efficiency and persistence of pro-regenerative signal transmission (96). Multiple independent research groups show inter-laboratory variations in the intensity and duration of conditioning injury effects, suggesting multi-factor regulation including experimental conditions, animal strains, and housing environments. Beyond experimental conditions, cross-species differences in optic nerve anatomy, RGC populations, and injury responses across rodents, larger mammals, and primates must also be considered when translating findings to human disease (97–100).Clinical translation faces challenges including time window restrictions (preconditioning must occur 1–2 weeks before injury), safety concerns regarding the active induction of inflammation, and the lack of standardized protocols (101–103).Recent experimental advances have demonstrated that fibronectin-derived peptides, sufficiently small for intraocular injection and optic nerve penetration, dramatically enhance nerve cell survival and axonal regrowth; when combined with gene therapy, regenerating axons reach the optic chiasm within 6 weeks post-injury (104). Comprehensive reviews emphasize that multi-therapeutic interventions targeting intrinsic growth programs, extrinsic inhibitory factors, and inflammatory microenvironment optimization show particular promise for achieving clinically meaningful regeneration over the extended distances required in human optic nerves (105).

### 4.2 Molecular integration of inflammatory signals and intrinsic regeneration programs

Inflammatory mediators released by microglia regulate RGC intrinsic regenerative capacity through multi-level molecular networks. OCM, as a key pro-regenerative molecule, binds to the RGC surface receptor ArmC10 with high affinity (dissociation constant approximately 30 nM), and its axon growth-promoting activity significantly surpasses that of classical neurotrophic factors BDNF, CNTF, and GDNF (89). Although the downstream signal transduction mechanisms of the OCM–ArmC10 axis remain incompletely elucidated, existing evidence indicates that the pathway converges on intracellular key growth regulatory nodes.

The activation of intrinsic regeneration programs by inflammatory signals occurs primarily through the relief of negative regulation. Under physiological conditions, PTEN suppresses PI3K–AKT–mTOR signaling through its lipid phosphatase activity (by dephosphorylating PIP3), while SOCS3 inhibits neuronal growth primarily through suppression of JAK-STAT3 signaling. Both pathways converge to maintain the low-growth state of adult neurons. Moderate inflammatory stimulation downregulates PTEN and SOCS3 expression or activity: PTEN downregulation releases mTOR from direct negative regulation, elevating the phosphorylation of downstream effectors S6K1 and 4E-BP1, while SOCS3 reduction enhances STAT3-mediated transcription of growth-associated genes (including Gap43 and Sprr1a). These parallel mechanisms synergistically initiate regeneration programs including protein synthesis, cytoskeletal reorganization, and growth-related gene expression (106). This mechanism explains why simple deletion of PTEN or SOCS3 promotes axonal regeneration, while inflammatory signals provide physiological pathways for the endogenous regulation of these inhibitory molecules. Fine-tuning of the microglial phenotype proves crucial for pro-regenerative effects; the C-type lectin receptor Dectin-1 plays a unique role in shaping pro-regenerative inflammatory microenvironments by initiating Syk–Card9 signaling, inducing microglial expression of pro-regenerative factors (IGF-1, GDNF) while suppressing the production of pro-inflammatory cytokines (TNF-α, IL-1β) (107). This signal selectivity suggests that through specific pattern recognition receptor activation, it may be possible to separate pro-regenerative effects from excessive inflammatory damage, thereby providing potential targets for precision regulation of microglial phenotype. Beyond extrinsic inflammatory signals, intrinsic heterogeneity among RGCs significantly influences regenerative outcomes. Alpha-RGCs, which comprise approximately 4% of total RGCs, exhibit markedly superior axon regeneration compared to other subtypes, attributed to higher baseline mTOR activity and increased expression of regeneration-associated genes, including osteopontin and GAP-43. This subtype-specific variation underscores the importance of considering RGC identity when designing microglial modulation strategies (108).

### 4.3 Phase-based inflammatory intervention strategies

Direct characterization of microglial dynamics following optic nerve crush has revealed distinct temporal phases of activation and phagocytosis (109). The regulatory roles of microglia in axonal regeneration exhibit significant time-dependence. Combining this with the spatiotemporal evolution patterns of neuroinflammation, post-injury microglial function can be divided into three consecutive stages: the early stage (1–3 days) during which microglia primarily execute phagocytic clearance functions and excessive activation suppression may potentially delay necrotic debris clearance; the transition stage (3–7 days) representing a critical phenotypic polarization window during which microglia exist in a dynamic balance between pro-inflammatory and potentially pro-repair functions; and the maintenance stage (entering the subacute phase after 7 days) during which microglia maintain relatively stable activation states, but pro-regenerative capacity gradually weakens and may progress toward chronification with improper inflammation regulation (110).

Intervention studies targeting these temporal characteristics reveal the therapeutic potential of precision regulation. Arg2 gene knockout significantly suppresses excessive microglial activation, inducing obvious axonal regeneration and protecting damaged RGCs on day 14 post-injury, indicating that regulating Arg2 activity during the subacute phase (7–28 days) holds promise for optimizing the pro-regenerative functions of microglia (111). Studies on the polyamine spermidine further emphasize the value of early intervention; continuous spermidine administration from 4 days before injury to 14 days post-injury significantly reduced RGC death and promoted axonal regeneration on days 7 and 14 post-injury, with the mechanisms involving spermidine’s effective suppression of microglial iNOS expression and chemokine (MCP-1, RANTES) production on days 4–5 post-injury (corresponding to the transition stage), thereby limiting inflammatory spread during the phenotypic transition window (112). *In vitro* experiments indicate that spermidine requires 1-hour pretreatment before inflammatory stimulation for optimal effects (113), highlighting the importance of preventive intervention while providing experimental evidence for determining clinical treatment timing.

## 5 Microglial inflammatory regulatory networks in myelin repair

Microglia play pivotal regulatory roles in myelin repair processes through mechanisms involving the recognition of endogenous danger signals, dynamic M1/M2 polarization switching, and coordination with other cell types. Myelin damage releases endogenous danger-associated molecular patterns (DAMPs) that sustain chronic inflammatory states, with pro-inflammatory microglia clearing inhibitory myelin debris during the acute phases and pro-repair microglia promoting oligodendrocyte progenitor cell (OPC) proliferation and differentiation. This repair process requires the coordinated cooperation of multiple cell types, including astrocytes and the vascular system.

### 5.1 Inflammatory response characteristics of myelin damage

Myelin damage resulting from optic nerve injury releases abundant myelin debris and myelin-associated inhibitory molecules that act as endogenous DAMPs, continuously activating microglia and maintaining chronic neuroinflammatory states (114). Molecules present in myelin debris, such as myelin-associated glycoprotein (MAG), oligodendrocyte myelin glycoprotein (OMgp), and Nogo proteins, not only inhibit axonal growth but also perpetuate pro-inflammatory activation of microglia through toll-like receptor (TLR) activation (115). Oligodendrocyte death further releases endogenous danger signals, including intracellular contents, DNA fragments, and organelle debris, which trigger additional inflammatory cascades upon recognition by pattern recognition receptors. This leads to a pathological cycle of myelin damage, inflammation activation, and further myelin injury.

### 5.2 Differential regulation of myelin regeneration by microglial polarization

M1 and M2 microglia play distinct roles in regulating myelin regeneration. During the acute phases of demyelinating injury, Pro-inflammatory microglia perform essential clearance functions by phagocytosing inhibitory myelin debris, thereby creating favorable conditions for subsequent repair processes. In demyelinating mouse models, Microglia with predominantly pro-inflammatory phenotypes dominate the early stages of disease, when oligodendrocyte progenitors (OPCs) are recruited and proliferate at lesion sites (116). However, persistent M1 activation leads to the secretion of pro-inflammatory factors that inhibit OPC proliferation and differentiation. A failure of myelin regeneration following optic nerve crush can be attributed to sustained microglial activation 21 days post-injury, which maintains a pro-inflammatory environment that impedes myelin repair (117).

In contrast, microglia with pro-repair characteristics play a positive regulatory role in myelin repair (118). As OPCs differentiate into mature oligodendrocytes, microglia transition to M2-like phenotypes, expressing markers such as Arg1, CD206, and IGF-1. Growth factors secreted by pro-repair microglia, including IGF-1, PDGF-AA, and FGF-2, promote OPC proliferation and maintain cell survival, while the release of anti-inflammatory factors like IL-10 and TGF-β helps create a local microenvironment conducive to OPC differentiation. Research indicates that delayed clearance of activated microglia after optic nerve crush enhances OPC maturation and differentiation into functional oligodendrocytes, facilitating effective myelin regeneration (119). These findings highlight that the timely switching of microglial polarization from M1 to M2 is crucial for successful myelin regeneration.

### 5.3 Multicellular collaborative networks in myelin repair

Myelin regeneration requires coordinated action from multiple cell types, including microglia, astrocytes, OPCs, and vascular endothelial cells (120). Astrocyte activation states significantly influence the outcomes of myelin repair: A2 astrocytes secrete pro-regenerative factors such as cholesterol and lactate, which provide metabolic support for oligodendrocyte function and myelin synthesis (121, 122). In contrast, A1 astrocytes primarily release inhibitory factors that impede myelin repair. The vascular system also contributes to the inflammatory regulation of myelin regeneration, with OPCs migrating along vascular structures to injury sites. Factors secreted by vascular endothelial cells, such as PDGF-AA, support OPC proliferation and survival (123).

Microglia influence the inflammatory microenvironment of myelin repair indirectly by regulating vascular permeability, modulating endothelial cell function, and maintaining blood-brain barrier (BBB) integrity. Based on existing evidence, therapeutic strategies that promote M2 microglial polarization may enhance myelin regeneration (124).

## 6 Therapeutic strategies and clinical translation prospects

**Figure 4:**
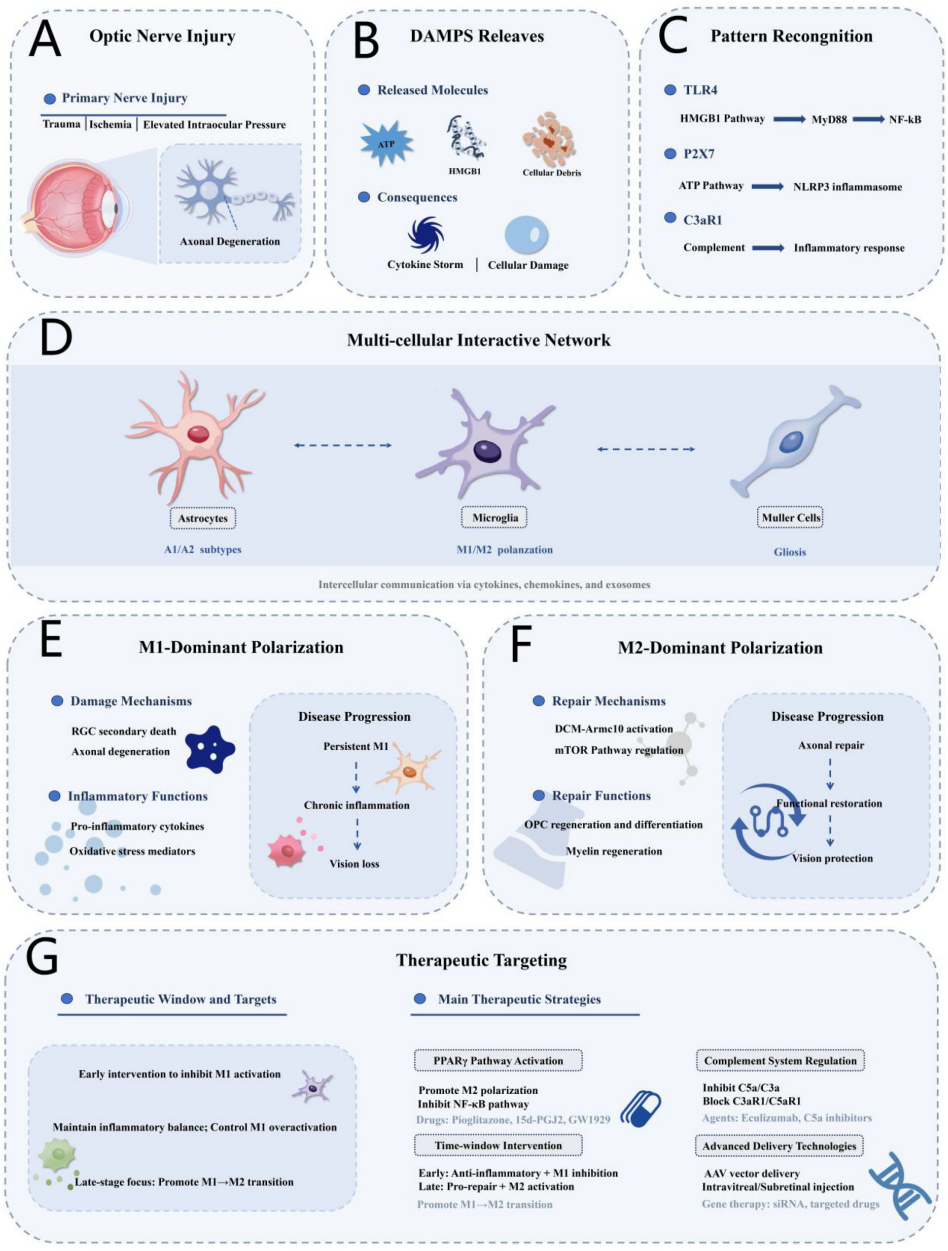
Comprehensive framework of neuroinflammatory mechanisms and therapeutic targeting strategies in optic nerve injury. **(A)** Optic Nerve Injury: Primary nerve injury caused by trauma, ischemia, or elevated intraocular pressure leads to axonal degeneration. **(B)** DAMPs Release: Injury triggers release of ATP, HMGB1, and cellular debris, resulting in cytokine storm and cellular damage. **(C)** Pattern Recognition: DAMPs activate TLR4 (HMGB1-MyD88-NF-κB pathway), P2X7 (ATP-NLRP3 inflammasome pathway), and C3aR1 (complement-mediated inflammatory response). **(D)** Multi-cellular Interactive Network: Astrocytes (A1/A2 subtypes), microglia (M1/M2 polarization), and Müller cells (gliosis) communicate via cytokines, chemokines, and exosomes. **(E)** M1-Dominant Polarization: Damage mechanisms include RGC secondary death and axonal degeneration. Inflammatory functions produce pro-inflammatory cytokines and oxidative stress mediators, leading to persistent M1, chronic inflammation, and vision loss. **(F)** M2-Dominant Polarization: Repair mechanisms include DCM-Armc10 activation and mTOR pathway regulation. Repair functions comprise OPC regeneration and differentiation and myelin regeneration, resulting in axonal repair, functional restoration, and vision protection. **(G)** Therapeutic Targeting: Strategies include PPARγ pathway activation (pioglitazone, 15d-PGJ2, GW1929), complement system regulation (eculizumab, C5a inhibitors), time-window intervention (early: anti-inflammatory + M1 inhibition; late: pro-repair + M2 activation), and advanced delivery technologies (AAV vectors, intravitreal/subretinal injection, gene therapy).

With advancing research on microglial M1/M2 polarization regulatory mechanisms, neuroinflammation treatment for optic nerve injury is entering a critical period of clinical translation. Building on temporal intervention principles, the PPARγ pathway, minocycline, and the complement system have emerged as therapeutic targets. Additionally, nanodelivery systems and AAV vector technologies offer new avenues for precision drug delivery, while advanced technologies such as single-cell analysis and molecular imaging are accelerating the development of precision monitoring techniques. Individualized treatment strategies and multimodal combination regimens show promising prospects. **Figure 4** provides a comprehensive framework integrating the pathophysiological mechanisms of optic nerve injury-induced neuroinflammation with corresponding therapeutic targeting strategies across different injury phases and cellular networks. This systematic overview synthesizes the molecular mechanisms of DAMP release, pattern recognition pathways, multicellular inflammatory networks, and therapeutic windows for intervention, offering a roadmap for translating mechanistic insights into clinical applications (**Table 1**).

**Table 1:** Therapeutic strategies targeting microglial inflammatory regulation in optic nerve injury.

Strategy	Mechanism of action	Preclinical evidence	Limitations	Development stage	Key references
PPARγ Activation	Promotes M2 microglial polarization through transcriptional regulation	Reduces hypoxic injury; neuroprotective in acute models	Optimal timing unclear; long-term efficacy in chronic conditions unvalidated	Preclinical	Zhao et al. (126)
Minocycline	Selectively inhibits M1 polarization; increases ARG1+ cell proportion	Delays RGC death in experimental glaucoma and optic nerve transection models	Non-selective; may block beneficial M2 functions; Phase II trial failed in AMD	Clinical trial (Phase II failed)	Keenan et al. (129)
Complement C5 Inhibition (Eculizumab)	Blocks C5 activation; reduces membrane attack complex formation	Reduced relapse rates in AQP4+ neuromyelitis optica	Infection risk (meningococcal); high cost; may interfere with physiological complement functions	FDA-approved (NMO)	Palace et al. (130)
Complement C1q Inhibition (ANX007)	Blocks classical complement cascade initiation	Phase I trials demonstrate safety and target engagement in glaucoma patients	Long-term efficacy unknown; repeated injections required	Clinical trial (Phase I)	Williams et al. (71)
Nanodelivery Systems (PLGA, Lipid NPs)	Targeted drug delivery across blood-retinal barrier; controlled release	Enhanced bioavailability; reduced systemic toxicity	Manufacturing complexity; long-term safety unknown	Preclinical	You et al. (131)
Polydopamine Nanoparticles	ROS scavenging; protects endothelial and neuronal cells; inhibits microglial activation	Single injection attenuates RGC loss; synergistic with brimonidine for axon regeneration	Biocompatibility requires long-term validation	Preclinical	Lou et al. (132)
Spermidine	Suppresses iNOS and chemokine production during transition phase	Natural compound; favorable safety profile	Narrow therapeutic window; requires pretreatment	Preclinical	Noro et al. (112)
Arg1 Modulation	Regulates microglial activation in subacute phase	Promotes axon regeneration; protects RGCs	Mechanism incompletely understood	Preclinical	Fouda et al. (111)
Aldose Reductase Inhibition (Sorbinil)	Inhibits AR-mediated inflammatory response in retinal microglia	Attenuates microglial activation and migration; promotes RGC survival after optic nerve crush	Specificity to retinal tissue requires validation	Preclinical	Wang et al. (147)
GPR84 Inhibition	Blocks rho-ROCK pathway activation in microglial subpopulation	Knockout prevents RGC loss; reduces TNF-α and IL-1α expression	Mechanism requires further characterization	Preclinical	Sato et al. (37)
Fibronectin Peptides	Promotes nerve cell survival and axon regeneration	Reaches optic chiasm within 6 weeks; synergistic with gene therapy	Optimal dosing and combination protocols need refinement	Preclinical	Lukomska et al. (104)

### 6.1 Development and challenges of strategies based on microglial function modulation

An important consideration for therapeutic translation is that complete microglial elimination may not be beneficial. Studies have used pharmacological CSF1R inhibition to deplete microglia in optic nerve crush models. Despite efficient microglial removal, RGC degeneration continued unaltered (125). Additionally, axon regeneration remained unaffected. These findings carry important therapeutic implications. Rather than pursuing complete microglial elimination, therapeutic strategies should focus on modulating microglial polarization and function. Complete depletion may inadvertently remove both detrimental and beneficial microglial activities. Therapeutic strategies based on microglial function modulation represent a shift from traditional broad suppression to precision temporal regulation. This approach builds on an understanding of the spatiotemporal dynamics of inflammatory responses, ideally maintaining moderate pro-inflammatory activation during the acute phase for effective injury clearance, promoting transition to pro-repair phenotypes during the subacute phase, and maintaining long-term M2 stability during the chronic phase. However, clinical translation faces challenges such as individual differences, disease heterogeneity, and determining precise therapeutic time windows.

PPARγ signaling pathway activation strategies illustrate this complexity. The PPARγ agonist 15d-PGJ2 promotes microglial polarization toward pro-repair phenotypes and provides neuroprotective effects in hypoxic injury models (126), but research has predominantly focused on acute injuries, with long-term effects in chronic neurodegenerative diseases like glaucoma remaining insufficiently validated. Timing selection remains a critical challenge: premature activation may interfere with pro-inflammatory microglia-mediated clearance, while delayed activation may miss the optimal neuroprotective window. The clinical development of microglial inhibitors has demonstrated significant discrepancies between animal models and clinical trials. Minocycline, a well-known inhibitor, showed neuroprotective effects in animal models by delaying RGC death after experimental glaucoma and optic nerve transection (127). Minocycline’s mechanism involves selective inhibition of LPS-induced pro-inflammatory polarization, increasing the proportion of ARG1-positive cells from 14–16% to 45–51% in retinal ischemia-reperfusion models (128). However, a Phase II randomized controlled trial targeting age-related macular degeneration failed to demonstrate expected efficacy (129), reflecting minocycline’s insufficient functional selectivity. While inhibiting harmful pro-inflammatory responses, it also blocks beneficial pro-repair functions, potentially nullifying therapeutic effects.

Precision modulation of the complement system represents a promising but more complex therapeutic strategy. The C5 inhibitor eculizumab significantly reduced relapse rates and improved functional outcomes in patients with AQP4-positive neuromyelitis optica spectrum disorders (130), though long-term use raises safety concerns, particularly regarding increased susceptibility to infections like meningococcal infection. Complete blockade of the complement system may interfere with its physiological functions in neural development, synaptic pruning, and cellular debris clearance (70). Ideal complement modulation strategies should selectively inhibit pathological overactivation while preserving physiological protective functions, requiring a more nuanced understanding of complement system mechanisms at different disease stages and in different tissue environments.

### 6.2 Precision delivery and monitoring technologies

The blood-retinal barrier (BRB) poses a significant obstacle to the effective delivery of anti-inflammatory drugs to damaged optic nerve tissue. Advanced nanomedicine delivery systems have achieved selective BRB penetration by optimizing particle size distribution, surface charge properties, and hydrophilic-hydrophobic balance, specifically targeting activated microglia (131). Lipid nanoparticle drug carriers protect anti-inflammatory active ingredients from degradation by *in vivo* enzymes, maintaining therapeutic concentrations at inflammatory sites through controlled-release mechanisms and enabling precise regulation of microglial polarization between pro-inflammatory and pro-repair states. Recent studies have established polydopamine nanoparticles as versatile therapeutic platforms that efficiently eliminate reactive oxygen species (ROS), protect endothelial and neuronal cells from oxidative damage, and inhibit microglial activation. A single intravitreal injection significantly reduces RGC loss by eliminating ROS and reducing inflammatory responses (132). When loaded with brimonidine, these nanoparticles promote axon regeneration and restore visual function. Comprehensive reviews highlight emerging non-invasive delivery modalities, theranostic agents, and gene therapy vectors as next-generation approaches that could revolutionize retinal neurodegeneration treatment.

Serotype optimization of AAV vector technology enhances transduction specificity for retinal cell types, with novel vectors like AAV2/2 and AAV2/8 demonstrating excellent gene delivery efficiency to microglia and RGCs, effectively delivering anti-inflammatory genes or key polarization-regulating transcription factors like PPARγ and KLF4 (133). However, these advanced delivery technologies remain largely experimental, with safety and efficacy still requiring long-term clinical validation. The potential toxicity of nanodrug carrier systems and the immunogenicity of AAV vectors must also be further investigated (134, 135).

The rapid development of single-cell analysis technology is transforming the precision diagnosis and dynamic monitoring of post-injury neuroinflammation. Single-cell mass cytometry (CyTOF) can identify over 40 surface markers at the single-cell level, allowing for fine-grained analysis of microglial activation states and polarization phenotypes (55, 136). Single-cell transcriptomics has identified functionally specific microglial subpopulations and molecular signatures associated with various stages of optic nerve injury (27). Methodological improvements in retinal single-cell RNA sequencing, along with large-scale atlases of retinal cells, now provide unprecedented resolution for characterizing microglial-RGC interactions across development and degeneration (137, 138). TSPO-PET molecular imaging allows non-invasive, real-time monitoring of cellular activation states through detection of the 18 kDa translocator protein expressed specifically by microglia, aiding in the assessment of temporal inflammation evolution post-injury (139). The development of multiparameter optical coherence tomography (OCT) imaging technology enables high-resolution monitoring of retinal microstructural changes at the subcellular level, detecting inflammation-related alterations in nerve fiber layer thickness and tissue edema (140). A particularly promising imaging approach is the Detection of Apoptosing Retinal Cells (DARC), which enables direct visualization of individual dying RGCs *in vivo* using fluorescently labeled Annexin V that binds to phosphatidylserine exposed on apoptotic cells. Clinical trials have shown that DARC can detect RGC apoptosis months before structural changes appear on OCT or functional losses manifest in visual fields, providing an early window for therapeutic intervention (141–143). Recent advances have integrated artificial intelligence algorithms to automate DARC quantification, enhancing reproducibility (144). Furthermore, DARC’s ability to simultaneously image immune cell populations offers insights into neuroinflammation-neurodegeneration coupling, positioning this technology as both a biomarker for patient stratification and an outcome measure for clinical trials.

### 6.3 Individualized treatment strategies and clinical translation barriers

The clinical translation of individualized neuroinflammatory treatment for optic nerve injury faces fundamental challenges despite precision medicine’s success in other fields (145, 146). A critical barrier is the lack of reliable biomarker systems. While serum inflammatory ratios such as TNF-α/IL-10 show research potential for assessing disease status and predicting treatment responses (147, 148), their clinical utility is severely compromised by poor standardization and reproducibility due to variability in sample collection timing, processing methods, storage conditions, and detection platforms (149, 150). Similarly, although gene polymorphism analysis offers pharmacogenomic insights—such as NF-κB pathway polymorphisms influencing anti-TNF efficacy in inflammatory bowel disease (151) and PPARγ variants affecting agonist responses (152)—neuroinflammation’s multifactorial nature limits single-gene predictive power. Comprehensive approaches using polygenic risk scores and systems biology may provide greater utility but entail substantial costs and interpretative complexity. Establishing internationally recognized standards and quality control systems is therefore essential for advancing individualized treatment from research to clinical practice.

Beyond biomarker challenges, therapeutic implementation faces significant obstacles in trial design and outcome assessment. Multimodal combination therapies, exemplified by the synergistic effects of OCM with cAMP analogs on axonal regeneration (153) and rational neuroprotective-anti-inflammatory combinations that improve the inflammatory microenvironment (154), theoretically offer broader target coverage but require complex optimization of dose ratios, administration timing, drug interaction monitoring, and safety protocols, substantially increasing development costs. Most critically, traditional functional measures such as visual acuity and visual fields lack sensitivity to detect early neuroprotective effects or subtle improvements (155), risking misclassification of effective treatments in clinical trials. While emerging multimodal imaging and electrophysiological techniques provide more sensitive tools, their standardization and cost-effectiveness require validation. The relatively low incidence of traumatic optic nerve injury further impedes adequately powered randomized controlled trials (156), with current projections suggesting microglial modulation therapies may require 5–10 additional years before achieving widespread clinical application (157).

## 7 Conclusions

The neuroinflammatory regulatory role of microglia in optic nerve injury is highly complex and condition-dependent. Following injury, microglia are involved in critical pathophysiological processes, including injury clearance, axonal regeneration, and myelin repair, through mechanisms such as DAMP recognition, switching between pro-inflammatory and pro-repair phenotypes, and interactions within multicellular networks. The dual nature of microglial functions—where neuroinflammation can both exacerbate tissue damage and, under certain conditions, promote neural repair—has prompted a shift from traditional broad-spectrum anti-inflammatory approaches toward precision functional modulation.

Existing research has identified potential therapeutic targets, including PPARγ pathway activation, selective complement system intervention, and time-window-dependent modulation, providing a foundation for the application of advanced approaches such as nanodelivery technologies, single-cell analysis, and molecular imaging in precision diagnosis and therapy. However, substantial challenges remain in translating these findings into clinical applications. Key obstacles include fundamental differences between animal models and human diseases in terms of anatomical structure, cellular composition, and regenerative potential, which limit the translational value of animal research; the heterogeneity and dynamic evolution of microglial functional states, which complicate precision intervention; the strict time window requirements of conditioning injury effects, which often conflict with clinical practice; and the unclear mechanisms by which individual factors, such as age, genetic background, and disease state, influence treatment responses.

Future research should focus on developing model systems that more closely mimic human disease characteristics, utilizing single-cell multi-omics technologies to uncover the molecular basis of microglial functional heterogeneity, and advancing individualized treatment strategies based on patient stratification. Additionally, refining biomarker detection platforms and efficacy evaluation systems is critical. With ongoing technological progress and a deeper understanding of underlying mechanisms, therapeutic strategies that modulate microglial function hold promise for providing clinically meaningful visual function protection and restoration in patients with optic nerve injury. These approaches may also lay the groundwork for immunomodulatory therapies in other central nervous system diseases. While the path from bench to bedside remains challenging, recent advances offer reasons for optimism that precision neuroinflammation modulation will ultimately fulfill its therapeutic potential.
